# A New Design for Reference Values Assignment in Proficiency Testing for Fat and Crude Protein in Raw Milk for a Limited Number of Participants

**DOI:** 10.3390/foods13172693

**Published:** 2024-08-26

**Authors:** Susan Poo, Miguel Palma, Ociel Muñoz

**Affiliations:** 1 Laboratory for Measurement Quality Assurance (LACM), Institute of Food Sciences and Technology, Faculty of Agricultural and Food Sciences, Universidad Austral de Chile, Valdivia 5090000, Chile; susan.poo@uach.cl; 2 Department of Analytical Chemistry, Center of Agri-Food and Wine Research (IVAGRO), Faculty of Science, University of Cadiz, 11510 Puerto Real, Spain; 3 Institute of Food Sciences and Technology, Faculty of Agricultural and Food Sciences, Universidad Austral de Chile, Valdivia 5090000, Chile; ocielmunoz@uach.cl

**Keywords:** proficiency testing, reference value, assigned value, reduced number of laboratories, raw milk, fat, crude protein

## Abstract

Proficiency testing (PT) allows food laboratories to endorse their competency to provide food safety guarantees to producers and consumers. One of the recommended methods for assigning reference values in PT with a small number of participants consists in considering the results that a laboratory obtains by means of a calibration test based on certified reference material (CRM). The present study delves into the results from eight PT rounds on the determination of fat and crude protein from raw milk, with modifications in the number of samples and the analysis sessions from that required by the ISO 13528:2022. The uncertainty criterion of the assigned value established by the ISO 13528:2022 standard was met by 93% of the participating laboratories, which allowed most participants to be evaluated through z-score. The assigned values were generally compatible with the results obtained by the participants. Thus, it can be concluded that the design for the assignment of the reference value is appropriate for PT with a limited number of participants. It is recommended for future PT to limit the uncertainty of the CRM according to their availability and to update the standard deviation of the proficiency assessment for the Mid-Infrared Spectroscopy method (MIR).

## 1. Introduction

The reliability of analytical data is an essential factor for decision-making processes in the food industry, so that the quality and safety of food products can be guaranteed. For this reason, there is a growing need to provide reliable analytical results. This has led to the implementation of laboratory management systems and has encouraged the seeking of accreditations under specific standards such as ISO/IEC 17025:2017 [1]. This has resulted in the development of new insights in areas such as validation or verification of methods, uncertainty assessment, control charts, reference materials, proficiency testing and others [2].

Proficiency testing (PT) is the evaluation of participants’ performance against pre-established criteria by means of interlaboratory comparisons [3]. It is, therefore, a tool for corroborating the competency of testing laboratories [4,5] and a requirement for laboratories accredited with the ISO/IEC 17025:2017 standard [1]. For this reason, the efficiency of the performance evaluation is of the utmost interest both for participants and for the accrediting entities [6]. The stages of a PT comprise design and planning, preparation and distribution of PT items, data analysis, evaluation of the participants’ performance and a final report. A PT item is defined as “sample, product, artefact, reference material, piece of equipment, measurement standard, object, image, data set or other information used for the proficiency testing” [3]. A critical aspect of data analysis is the assignment of the reference value or assigned value, which is defined as the “value attributed to a particular property or characteristic of a proficiency testing item” [3], against which the results from the participants are compared in order to evaluate their performance. Different approaches or procedures for the assignment of the reference value are described in certain protocols and international standards, such as ISO 13528:2022 [7], ISO/IEC 17043:2023 [3] or the IUPAC harmonized protocol for the proficiency testing of chemical analysis laboratories [8].

The dairy sector represents one of the most important agrifood chains in the economy of many countries and is particularly relevant for Chile’s economy [9], which relies on thousands of producers that provide dairy products to its population [10]. The validity of the results obtained from laboratory analyses is vitally important for a proper quality control that fulfills the regulatory requirements, reassures consumers and provides transparency in any dairy transaction. Some authors from different countries [11,12] have pointed out that the cost of participating in international PT, in addition to the time required for transport and customs clearance of the materials, underscores the need for PT suppliers from the same country where the PT is performed. A similar situation occurs in Chile, where laboratories employ mainly instrumental analytical methods whose results require verification by regularly participating in PT rounds organized by the Metrology Division of the Laboratory for Measurement Quality Assurance LACM^®^ in the Universidad Austral de Chile. The number of participants in these PTs ranges between eight and twenty, which is considered a “small” number according to the IUPAC-CITAC guidelines with regard to the “Selection and Use of Proficiency Testing Schemes for a Limited Number of Participants—Chemical Analytical Laboratories (IUPAC Technical Report)” [13]. PT with a large number of participants offer several alternatives for an appropriate evaluation of their performance [14]. However, for a small number of participants, the ISO 13528:2022 standard [7] recommends, whenever possible, assigning the reference value following a metrologically valid procedure that does not depend on the results obtained by the participants, as in the IUPAC/CITAC guide for the selection and use of PT for a limited number of participants [13].

On the other hand, the number of scientific articles referring to the implementation of different statistical approaches to PT with a small number of participants is rather limited. This has been pointed out by authors such as Olivares et al. [2] or Milde et al. [15], who highlight in their studies the importance of counting on PT assigned values that can be metrologically traced from the chemical point of view [16,17,18], as well as the benefits of using PT reference values for food matrices that are independent from the participants’ results, such as the use of a reference method or formulation [19,20,21]. However, it should be noted that for some PT providers, there might be certain technical difficulties such as the unavailability of appropriate certified reference materials (CRMs), experimental difficulties or high implementation costs [22,23]. On the other hand, there must be a procedure in place for the preparation of the PT items that allows the obtainment of a sufficiently homogeneous and stable material [3].

Because of the small number of participants, the PT provider, i.e., the Metrology Division of the LACM^®^ (Laboratory for Measurement Quality Assurance of the Austral University of Chile, Valdivia, Chile), determines the reference value for some of the PT based on the results obtained by a particular laboratory after performing a calibration test based on a (CRM), which is one of the methods described by the ISO 13528:2022 standard [7]. However, the design applied by this PT provider also contemplates a modification in the number of samples and incorporates other aspects, such as the suggestions by Maroto [24] and Schiller [25], which consist in incorporating the influence of the analytical session. The present study aims to validate this design while bearing in mind these modifications. For this purpose, our research will be based on the results achieved from the implementation of this design to a number of PT rounds held in Chile. This should allow us to determine if this design meets the standard uncertainty criterion for the assigned value as set out by the standard ISO 13528:2022 [7]. Otherwise, it will be necessary to incorporate new modifications. Since there is no evidence of the implementation of this design (with the aforementioned modifications) to other PT, its study is of interest to the agrifood sector, as it is a model that could be successfully applied when a small number of participating laboratories are involved.

## 2. Materials and Methods

This research was at the Metrology Division of the Laboratory for Measurement Quality Assurance LACM^®^, (according to its initials in Spanish, Laboratorio para el Aseguramiento de la Calidad de la Medición), which belongs to the Institute of Science and Food Technology, ICYTAL, (according to the initials of its Spanish name, Instituto de Ciencia y Tecnología de los Alimentos) at the Universidad Austral de Chile (Valdivia, Chile). This PT provider operates according to the requirements set out by the standard ISO 17043:2010 [26]).

### 2.1. Planning, Preparation and Distribution of the PT Items

The planning, preparation and distribution of the PT items to the participants and to the laboratory that performed the analyses to obtain the reference value were coordinated by the Metrology Division of the LACM^®^ according to their management procedures, which are based on the guidelines established by the ISO/IEC 17043:2010 standard [26]. The PT items were prepared using raw milk supplied by several dairy producers from Southern Chile (Región de Los Lagos and Región de Los Ríos). An amount of 0.1 g bronopol/100 g milk was used as a preservative. For each PT item, the raw milk was mixed and later dosed into polypropylene screw-capped vials. The mixing procedure, which ensured homogeneity between vials [27], was based on the expertise of the PT provider regarding the preparation of reference materials and PT items for dairy matrices. The sample vials were filled with 50 mL of the milk. For the assignment of the reference values based on a calibration test, CRMs from the German RM producers muva Kempten and DRRR were used. For the delivery of the PT items to the different laboratories, a packaging method that maintained the temperature of the PT items within a range of 1–8 °C during transport was employed. The number of laboratories that participated in the various PT rounds ranged from 12 to 19.

The homogeneity and stability of the PT items was verified by analyzing their fat and crude protein content using the Mid-Infrared Spectroscopy (MIR) method at the beginning and at the end of the analysis period of each PT round. These analyses were performed by a laboratory accredited with the ISO 17025:2017 standard [1]. Jointly with the assignment of the reference value, the reference methods of Röse Gottlieb [28] and Kjeldahl [29] to determine fat and crude protein content, respectively, were used to corroborate the homogeneity of the items. In order to compare the results, the criteria set out by the ISO 13528:2022 standard were applied [7], which confirmed that the material was sufficiently homogeneous and stable over the assay period. Given that these homogeneity and stability assays do not constitute the objective of this study, they have been mentioned herein just as preliminary aspects of the same.

### 2.2. Reference Value Assignment

#### 2.2.1. Experimental Design

The experimental design for the assignment of the reference value (assigned value) was based on one of the alternatives described in ISO 13528:2022 [7], Section 7.5.2, “Results from one laboratory”. This alternative consists in a single laboratory analyzing the PT item by using the reference method or another suitable method. This is also calibrated against a reference value obtained from a CRM of a very similar nature to that of the PT item. For the construction of the design, the formula proposed by the ISO 13528:2022 [7] standard was modified in order to allow a short number of samples (so that in can be used in PT with a limited number of participants). Furthermore, the suggestions from Maroto [24] and Schiller [25], which contemplate the analysis session as a source of error, were also taken into account. The experimental design consisted in the analysis of a vial of each PT item with one (1) replica for each one of the three analysis sessions, while for the CRM, 2 replicas were analyzed in each session. Each replica consists in the repetition of the analysis (under repeatable conditions), while an assay session can be defined as the whole set of analyses performed under repeatable conditions. The data of the experimental design are presented in Table 1.

#### 2.2.2. Data Source

The data from a total of 8 PT rounds of fat and crude protein in raw milk, held between 2017 and 2021 by the PT provider, were considered. The codes assigned to each round, as well as their completion date and the description of the CRM used, can be seen in Table 2. Six items were used for each PT round, so that a correlating number from 01 to 06 was assigned to each PT item alongside its assay identification code.

#### 2.2.3. Chemical Analysis for the Assignment of the Reference Value

The chemical analysis of the PT items and of the CRM to determine the reference value were performed by the Analytical Division of the LACM^®^/Universidad Austral de Chile. The “Röse-Gottlieb” method, based on the ISO 1211:2010(E) standard [28], and the Kjeldahl method, based on the ISO 8968-3:2014 [30], were used to determine the fat and protein content.

The reference value assigned to each PT item was calculated based on the results obtained through the laboratory analyses, which were performed according to the experimental design described in Section 2.2.1. Equation (1) below was followed to calculate the assigned value. This formula was obtained by rearranging the terms in the equation described in the ISO 13528:2022 standard [7] as shown below:

(1) x^=x¯PTI−x¯CRM−cCRM,

where

x^ is the reference value of the PT item,
$c_{CRM}$ is the reference value of the CRM,
${\overset{¯}{x}}_{PTI}$ is the mean of the results from the *n* replicas of the PT item and
${\overset{¯}{x}}_{CRM}$ is the mean of the results from the *n* replicas of the CRM.

Equation (1) represents the results from two assays: one that characterizes the PT item (PTI) ${\overset{¯}{x}}_{PTI}$ and another one that analyses a CRM ${\overset{¯}{x}}_{CRM}$. This parallel assay is known as the calibration, accuracy verification or traceability verification assay [24,31]. The expression between parenthesis is the bias estimate, and it is considered the correction factor for ${\overset{¯}{x}}_{PTI}$.

In order to determine if the assigned value through the calibration assay using the CRM is compatible with the results obtained by the participants as a whole, in the present study, we have calculated the robust mean of the results obtained by the participants who used the MIR method. For this purpose, Tukey’s biweight robust M-estimator was employed [32], and the difference with respect to the reference value was calculated using R control charts. The differences were compared against a set target based on the criteria set out in the ISO 13528:2022 standard [7] and according to the precision indexes of the analytical methods [33].

#### 2.2.4. Standard Uncertainty of the Assigned Value

Given that Equation (1) will be used to calculate the assigned value, and by applying the law of propagation of uncertainty [34], the uncertainty of the assigned value ux^ was calculated according to Equation (2):

(2) ux^=ux¯PTI2+ux¯CRM2+uCRM2,

where

- $u_{{\overset{¯}{x}}_{PTI}}$ is the standard uncertainty of the assayed PT item. For this source of uncertainty, and in order to facilitate the comparison between rounds, a common uncertainty for all the PT items analyzed in a round was estimated:

(3) $$u_{{\overset{¯}{x}}_{PTI}}^{2}=\frac{1}{n}(s_{session}^{2}+s_{r}^{2}+s_{sample}^{2}),$$

where *n* is the number of sessions, $s_{session}$ is the standard deviation between sessions (obtained through ANOVA, where “session” is a random factor), $s_{r}$ is the standard deviation of the repeatability and $s_{sample}$ is the standard deviation between vials. Given that the PT item must be sufficiently homogenous (which is verified through the homogeneity test), “sample” is not an uncertainty source, in a similar way as repeatability, given that just one single replica is analyzed per session. Therefore, Equation (4) below was used:

(4) $$u_{{\overset{¯}{x}}_{PTI}}^{2}=\frac{1}{n}(s_{session}^{2}).$$

- $u_{{\bar{x}}_{CRM}}$ is the standard uncertainty of the CRM assay, which is obtained as follows: If $x_{CRMlm}$ is the result obtained from session *l* and replica *m*, let *n* be the number of sessions and *q* the number of replicas in one session, then

(5) $${\overset{̿}{x}}_{CRM}=\frac{1}{nq}\sum_{l=1}^{n}\sum_{m=1}^{q}x_{CRMlm}.$$

The standard uncertainty of this mean can be calculated as:

(6) $$u_{{\bar{\bar{x}}}_{CRM}}=\sqrt{\left(\frac{s_{session}^{2}}{n}+\frac{s_{r}^{2}}{nq}\right)}$$

where $s_{session}$ and $s_{r}$ are obtained through an ANOVA, where “session” is considered a random factor. Similarly to the analysis of the PT item, the CRM must be sufficiently homogeneous; therefore, “vial” is not an uncertainty factor.

- $u_{CRM}$ is the CRM standard uncertainty, which is obtained based on the $U_{CRM}$ expanded uncertainty as set out in the CRM certificate, where $U_{CRM}=ku_{CRM}$ and k is the declared coverage factor.

The values obtained for the uncertainty of the assigned value were compared against the criterion established by the ISO standard 13528:2022 [7], according to which, the standard uncertainty of the assigned value ux^ can be neglected if the criterion ux^<0.3σ^ is met, where σ^ is the standard deviation for proficiency assessment used to evaluate the performance of the participants based on their z-score:

(7) $$z=\frac{x-\hat{x}}{\hat{\sigma }},$$

where x is the result obtained by the participating laboratory (mean of two replicas) and x^ is the assigned value. This criterion is set out because, if the standard uncertainty of the assigned value is rather large when compared against the criterion to evaluate the participants’ performance, there is a risk that some of the participants will receive an action or concern notice because the assigned value has not been determined with the required precision, which is not attributable to the participants’ performance. If the criterion is not met, i.e., if the standard uncertainty of the assigned value is not neglectable with respect to the standard deviation for proficiency assessment, then the PT provider may alternatively incorporate this uncertainty into the performance evaluation by considering the z′-score:

(8) $$z^{\prime }=\frac{x-\hat{x}}{\sqrt{{\hat{\sigma }}^{2}+{u_{\hat{x}}}^{2}}}.$$

The percentage of the PT items that met the uncertainty criterion of the assigned value was calculated for each PT round. On the other hand, the values obtained for the different uncertainty sources used for the calculation of the combined standard uncertainty of the assigned value were compared against the values estimated by the provider according to the design documentation [35].

### 2.3. Criteria to Evaluate the Performance of the Participants

The performance of the participants in a PT has been evaluated through the z-score (Equation (7)) or the z’-score (Equation (8)), as explained in Section 2.2.4. The participants’ identities and their results in the PT rounds have remained unidentified throughout this study in accordance with the ISO/IEC 17043:2023 standard [3]. Therefore, the data displayed herein are limited to exclusively those required to accomplish the objectives of this research.

The values of σ^ corresponding to the standard deviation used for the evaluation of the participants’ performance [35] were revised in order to determine if they continued to be suitable for the PT scheme when considering the current state of the art of the corresponding methods and standards. It should be born in mind that the σ^ values of the PT rounds held between 2017 and 2021 had been established according to the precision indexes of the analytical methods used by the participants. Such values do not depend on the results obtained by the participants and, therefore, remain invariable throughout the different PT rounds according to the criterion “fitness for purpose” [7,8]. The methods used by the participants to determine fat content were as follows: Röse-Gottlieb, based on the standard ISO 1211:2010(E) [28]; Gerber (described in the standard 19662:2018 [36]; and Mid-Infrared Spectroscopy or MIR (ISO 9622/IDF 141:2013 [37]). With regard to the determination of crude protein, the participating laboratories employed the following two methods: the Kjeldahl method, which is based on the standard ISO 8968-3/IDF 20 [30] and determines the nitrogen content to be multiplied by the factor 6.38 in order to convert it into the crude protein content; and Mid-Infrared Spectroscopy (MIR) [37]. The latter one was predominantly used by the participants to determine both fat and crude protein content in raw milk. The Röse-Gottlieb [28] and Kjeldahl [30] methods are also used as references for the calibration of the MIR methodology.

The method followed to determine the standard deviation for proficiency assessment σ^ according to the different analytical methods used by the participants is described below.

#### 2.3.1. Mid-Infrared Spectroscopy (MIR) (Fat and Crude Protein)

For the MIR method, σ^ was obtained based on the precision data in the ISO 8196-3 standard from 2009 [38], where the standard deviation of the intralaboratory reproducibility $\sigma_{Rintra}$ is 0.028g fat or crude protein/100 g milk and the standard deviation of the repeatability $\sigma_{r}$ is 0.014g fat or crude protein/100 g milk, for fat content levels in the range 2.0 to 6.0 g/100g and for crude protein content levels between 2.5 and 4.5 g/100 g. On the other hand, the standard deviation of the accuracy $\sigma_{yx}$ either for fat or protein is 0.070 g/100 g milk. According to the criterion mentioned in Part 2 of the standard (ISO 8196-2) [39], assuming accurately calibrated equipment, an instrumental error $e_{{\overset{¯}{x}}_{i}}$ encompasses both the precision and accuracy errors. It is therefore expected that the instrumental result from a sample subjected to the assay would be within a confidence range based on this $e_{{\overset{¯}{x}}_{i}}$ value and, for the same reason, it should be suitable to allow the evaluation of the participants’ performance.

Error $e_{{\overset{¯}{x}}_{i}}$ is given by the following expression (Equation (9)):

(9) $$e_{{\overset{¯}{x}}_{i}}=\pm {\left\{\left[\sigma_{R}^{2}-\left(1-\frac{1}{n}\right)\sigma_{r}^{2}\right]+\sigma_{yx}^{2}\right\}}^{1/2},$$

Equation (9) from the guidelines outlined in the ISO 8196-2 standard [39] was adopted, although it was simplified as it was assumed that the equipment had been properly calibrated, while part of the original expression corresponded to a theoretical calibration error roughly equal to 1. Thus, the above-mentioned precision values would result in an instrumental error $e_{{\overset{¯}{x}}_{i}}$ equal to 0.075 g fat or crude protein/100 g milk. Therefore, for the results of a single sample analyzed in duplicate using the instrumental MIR method, a σ^ value equal to 0.075 g fat or crude protein/100 g milk was established.

#### 2.3.2. The Röse Gottlieb Method (Fat)

For the Röse Gottlieb, Gerber and Kjeldahl methods, the equation described in Section 8.5.1 of the ISO 13528:2022 standard [7], based on the repeatability and reproducibility of an earlier collaborative study on a measuring method, was used:

(10) σ^=(σR2−σr2+σr2/m),

where

$\sigma_{R}$ is the standard deviation of the reproducibility of the analytical method,
$\sigma_{r}$ is the standard deviation of the repeatability of the analytical method and
*m* is the number of replicas performed by each laboratory participating in the PT.

For the Röse Gottlieb extraction method, the precision for whole milk set out by the ISO 1211:2010(E) standard “Milk—Determination of fat content—Gravimetric method (Reference method)” was used [28], where $\sigma_{R}$ = 0.020 g fat/100 g milk and $\sigma_{r}$ = 0.015 g fat/100 g milk. These indexes were obtained from a collaborative assay on whole milk samples with between 3.0 and 5.8 g fat/100 g milk. By applying Equation (10), σ^ = 0.017 g fat/100 g milk was obtained from a duplicate analysis.

#### 2.3.3. The Gerber Method (Fat)

In order to determine σ^ applicable to the Gerber method, the precision set out by the standard AOAC Official Method 2000.18 was considered [40] based on an interlaboratory assay. For raw milk, the standard deviation of the repeatability $\sigma_{r}$ is 0.023 g fat/100 g milk and the standard deviation of the reproducibility $\sigma_{R}$ equals 0.053 g fat/100 g milk. By applying Equation (10) to two replicas, the value obtained for σ^ was 0.050 g fat/100 g milk.

#### 2.3.4. The Kjeldahl Method (Crude Protein)

For the Kjeldahl method, the precision set out by the ISO 8968 standard, which comprises two parts [29,30], corresponding to the macro and the semi-micro block respectively, was considered. By applying Equation (10) and considering the precision indexes of the two parts in the above mentioned ISO standard, values between σ^ = 0.013 g crude protein/100 g milk and σ^ = 0.015 g crude protein/100 g milk were obtained. In order to avoid any limitations on the use of either of the alternative methods, the value for σ^ = 0.015 g crude protein/100 g milk was used, which is obtained from a repeatability equal to 0.038 g crude protein/100 g milk and a reproducibility equal to 0.049 g crude protein/100 g milk.

## 3. Results

### 3.1. Reference Values Assigned to the PT Items

The reference values assigned to each PT item (obtained from Equation (1)) ranged between 2.72 and 4.91 g fat/100 g milk and between 3.00 and 4.13 g crude protein/100 g milk. Both of these values are within the range established by the PT provider for the measurand content. Based on the expression between parentheses in Equation (1), the bias of the CRM could be determined as a single value for each PT round (mean of the three sessions). The results from each PT round are included in Appendix A (fat results are displayed in Table A1 and crude protein in Table A2). For the fat and the crude protein measurements, an average bias (based on all the PT rounds) of 0.0015 g fat/100 g and 0.0050 g crude protein/100 g were obtained, respectively. For both measurands, the average bias is below the value estimated by the PT provider (0.0029 g fat/100 g milk and 0.0120 g crude protein/100 g milk) in the design documentation [35], which was based on previous PT rounds. By applying the t Student statistical test, it could be inferred, at 95% confidence, that the average bias was not statistically different from zero for either of the measurands (*p*-value 0.6764).

**Figure 1:**
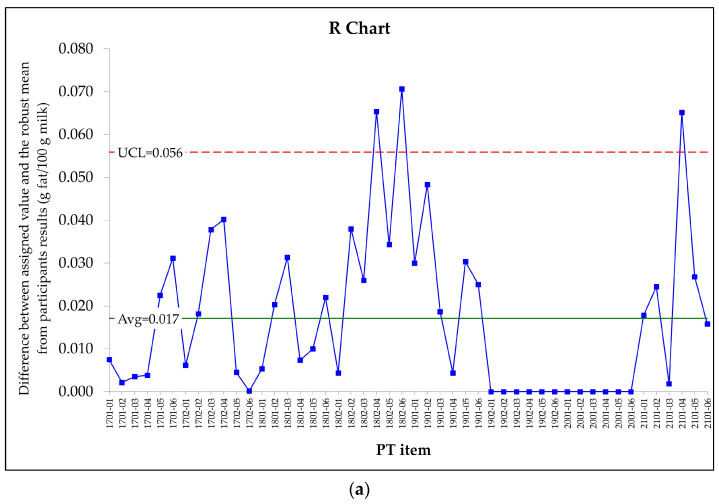

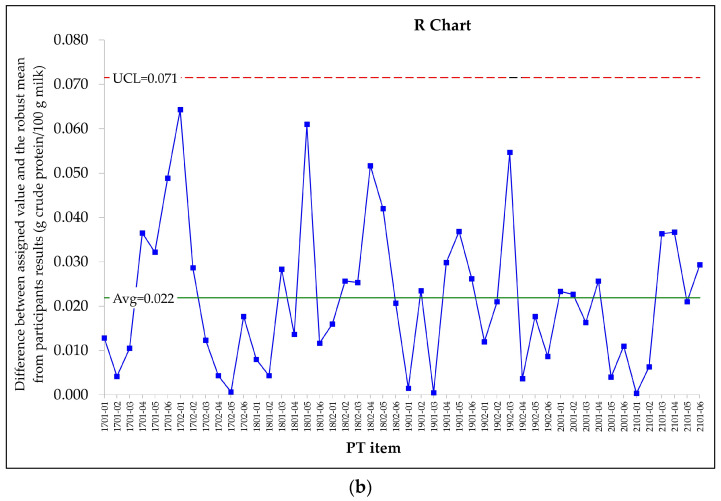
(**a**) R control chart showing the differences between the assigned value based on the CRM calibration assay and the robust mean obtained by the participants who applied the MIR method in the rounds conducted by the PT provider between 2017 and 2021 to determine the fat content in raw milk; (**b**) R control chart showing the differences between the assigned value based on the CRM calibration assay and the robust mean obtained by the participants who applied the MIR method in the rounds conducted by the PT provider between 2017 and 2021 to determine the crude protein content in raw milk. UCL = Upper Control Limit. Avg. = average.

According to the IUPAC/CITAC guide for PT with a small number of participants [13], a PT is successful when its outcome is compatible with the value that was assigned when using a CRM; this concept is also mentioned in the ISO standard 13528:2022 [7]. Several authors have investigated the differences between the values obtained through the independent methods used by the participants and the consensus value [6,17,18,41,42,43]. In order to determine if the value assigned to each PT through the calibration assay using the CRM is compatible with the results obtained by the participants as a whole, in the present study, we have calculated the difference between the reference values that had been assigned through the CRM calibration assay and the robust mean of the actual results obtained by the participants who used the MIR method. Those differences have been represented in Figure 1 below.

In relation to compatibility, Section 7.8 of the ISO 13528:2022 standard [7] describes a procedure to compare the consensual value against a value independent from the participants. It also points out that the difference between both results should not be greater than the expanded uncertainty $U_{dif}$ of the difference $x_{dif}=x^{*}$ − x^. The standard uncertainty is obtained as follows:

(11) udif=(ux∗2+ux^2),

where $x^{*}$ is the consensual robust mean of the participants, $u_{x^{*}}$ is the standard uncertainty of such mean, x^ is the reference value obtained independently and ux^ its standard uncertainty. The expanded uncertainty was obtained following the formula below:

(12) $$U_{dif}=ku_{dif},$$

where k is the coverage factor.

In order to establish a common target that can be used to compare the results obtained for the different PT items and rounds based on the concept “fitness for purpose”, the above-mentioned uncertainties are proposed to be based on the precision indexes of the analytical methods according to the equation set out in Section A.2.1 of the ISO 21748:2017(E) standard [33], which is based on the principle that “the reproducibility standard deviation obtained in a collaborative study is a valid basis for measurement uncertainty evaluation”. This equation can be seen below:

(13) $${u(y)}^{2}=s_{L}^{2}+s_{r}^{2},$$

where u(y) is the standard uncertainty of the analytical method, $s_{L}$ is the interlaboratory standard deviation (obtained from $s_{L}$ = $s_{R}^{2}-s_{r}^{2}$), $s_{R}$ is the standard deviation of the reproducibility and $s_{r}$ is the standard deviation of the repeatability that results from an interlaboratory study.

According to the precision indexes that have been pointed out in Section 2.3.1, Section 2.3.2, Section 2.3.3 and Section 2.3.4 and by applying the Equations (11)–(13), fat and crude protein target difference, i.e., $x_{dif}$ equal to 0.062 g fat/100 g milk and to 0.060 g crude protein/100 g milk, respectively, were obtained. These values were near the control limit obtained through the R control charts (Figure 1) and confirmed that the values assigned based on the CRM calibration assays were compatible with the robust mean obtained by the participants who employed the MIR method in 93.8% of the PT items for fat content and in 95.8% of the PT items for crude protein content.

#### 3.1.1. Combined Standard Uncertainty of the Assigned Values

**Figure 2:**
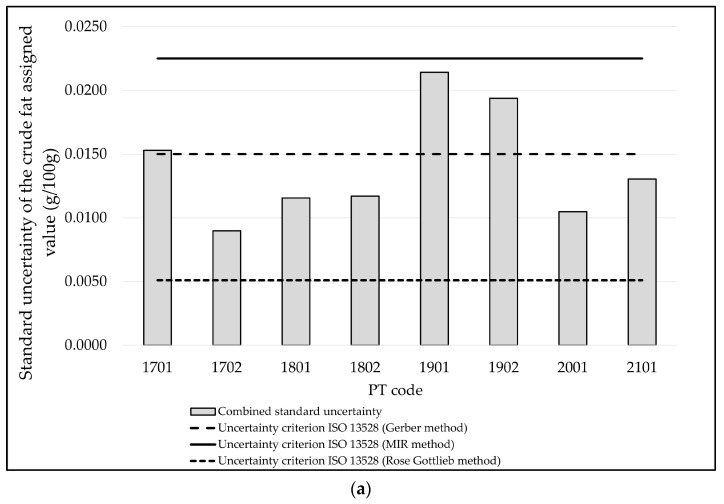

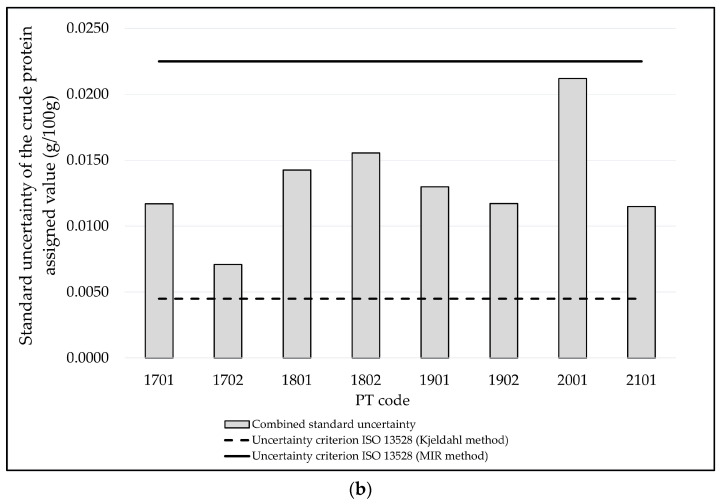
(**a**) Combined standard uncertainty obtained for fat from the PT rounds held by the PT provider between 2017 and 2021; (**b**) combined standard uncertainty obtained for crude protein from the PT rounds held by the PT provider between 2017 and 2021.

The combined standard uncertainty of the assigned values obtained by applying Equation (2) to each PT round to the fat and crude protein measurands are displayed in Figure 2.

It can be observed from Figure 2a,b that the criterion for the standard uncertainty of the assigned value following to the ISO 13528:2022 standard [7] is met in all the PT rounds by the laboratories that used the instrumental MIR method, both for fat and for crude protein, so that it is not necessary to incorporate the uncertainty for the performance evaluation, as a standard deviation for proficiency assessment σ^ equal to 0.075 g crude protein/100 g milk is considered.

On the other hand, with regard to fat, considering the σ^ of the Gerber method (0.050 g fat/100 g milk), the ISO 13528:2022 standard criterion [7] is met by 62.5% of the PT rounds, while the Röse Gottlieb does not fulfill the criterion in any of the rounds (with an σ^ equal to 0.017 g fat/100 g milk). It should be noted that most participants (93%) employed the MIR method, while the Gerber and the Röse Gottlieb methods were used just by 5% and 2% of them, respectively. However, in those cases where the criterion for uncertainty established by the standard ISO 13528:2022 [7] was not met, the performance of the participants could be evaluated by calculating their z’-score (Equation (8)).

The standard uncertainty criterion established by the ISO standard 13528:2022 [7] was not met in any of the PT rounds performed by those laboratories that used the Kjeldahl method to determine crude protein contents. It should be mentioned that most of the participants (92.5%) used the Mid-Infrared Spectroscopy or MIR method for this measurement and that just 7.5% of them used the Kjeldahl method. It must be born in mind that the standard deviation for proficiency assessment when the MIR method is used includes not only the precision indexes, but also the accuracy standard deviation [39], as explained in Section 2.3.1. On the other hand, similarly as for fat, in those cases, where the criterion set out by the ISO standard 13528:2022 [7] was not met, the performance of the participants could be assessed based on the calculation of their z’-score (Equation (8)).

#### 3.1.2. Uncertainty Components of the Assigned Values

The various uncertainty components of the assigned values were analyzed. The results regarding fat and crude protein are displayed in Table 3.

The main uncertainty sources are the uncertainty of the certified reference material (CRM) and the uncertainty of the PT item. With regard to the uncertainty of the CRM used in the design [35], it was estimated (according to the available CRMs), that the standard uncertainty $u_{CRM}$ could be as low or lower than 0.0070 g fat/100 g milk and 0.0075 g crude protein/100 g milk. However, it can be seen from the data in Table 3 that, from the EA GP LC 1801 onward, the standard uncertainty values of the CRMs are greater. According to the above-mentioned and based on the data in Table 3, if a CRM standard uncertainty value lower or equal to 0.018 g fat/100 g milk and 0.021 g crude protein/100 g milk is selected, the criterion for the uncertainty of the assigned value mentioned in the ISO 13528:2022 standard [7] should be met by the laboratories that used the MIR instrumental method, assuming that the other two uncertainty components are similar to those obtained from the PT rounds conducted between 2017 and 2021 (Table 3).

With respect to the standard uncertainty of the assay on the PT item $u_{{\overset{¯}{x}}_{PTI}}$, 12.5% of the PT rounds returned the value estimated by the design (i.e., equal to or lower than 0.0049 g fat/100 g milk) [35] regarding the fat, while for the crude protein, the estimated value (i.e., equal or lower than 0.0047 g crude protein/100 g milk) was obtained in all of the PT rounds. With regard to the other uncertainty component, which had been obtained from the CRM assay on fat $u_{{\overset{¯}{x}}_{CRM}}$, the value returned by the design (i.e., equal to or lower than 0.0040 g fat/100 g milk) was obtained [35] in 87.5% of the PT rounds, while for crude protein, a value equal to or lower than 0.0033 g crude protein/100 g milk was obtained in 50.0% of the PT rounds.

In certain cases, the values corresponding to the different sources of uncertainty are greater that the initially estimated values [35], although the combined standard uncertainty fulfills the ISO 13528:2022 standard criterion [7] in 93% of the cases (as pointed out in Section 3.2.1), so that the performance by the participants can be assessed according to their z-score or z’-score. Therefore, this design is considered to be suitable for the assessment of the participants’ performance based on a value independent from the participants and traceable by a larger group of expert laboratories by using CRM calibration assays for the assignment of the reference values.

### 3.2. Criteria for the Evaluation of the Participants’ Performance

The standard deviation values for the evaluation of the proficiency σ^ used for the evaluation of the performance (z-score and z’-score) in the PT rounds conducted between 2017 and 2021 were obtained based on the precision indexes of the analytical methods used by the participants, as already mentioned in Section 2.3. The state of the art of each one of the analytical methods used is described below.

#### 3.2.1. Instrumental MIR Method (Fat and Crude Protein)

According to the previously mentioned in Section 2.3.1, for the instrumental MIR method, σ^ was calculated based on the precision and accuracy data established by the ISO 8196-3 standard, 2009 version [38]. With regard to the current state of the art of the analytical method, a new version of the ISO 8196-3 standard, issued in 2022 [44], has been found. This version establishes a range between 2.0 and 6.0 g fat/100 g milk and between 2.5 and 4.5 g crude protein/100 g milk, a $\sigma_{Rintra}$ equal to 0.020 g fat or crude protein/100 g milk for filter instruments and 0.014 g fat or crude protein/100 g milk for instruments with Fourier-transform technology (FT). Given that for any given PT, either equipment type could be used, it is suggested that the greatest value should be used in future assays (i.e., 0.020 g fat or crude protein/100 g milk). With respect to $\sigma_{r}$, values equal to 0.014 g fat or crude protein/100 g milk, in the case of filter instruments, and to 0.008 g fat or crude protein/100 g milk, in the case of FT technology equipment, are mentioned. On the other hand, $\sigma_{yx}$ is equal to 0.050 g fat or crude protein/100 g milk.

**Figure 3:**
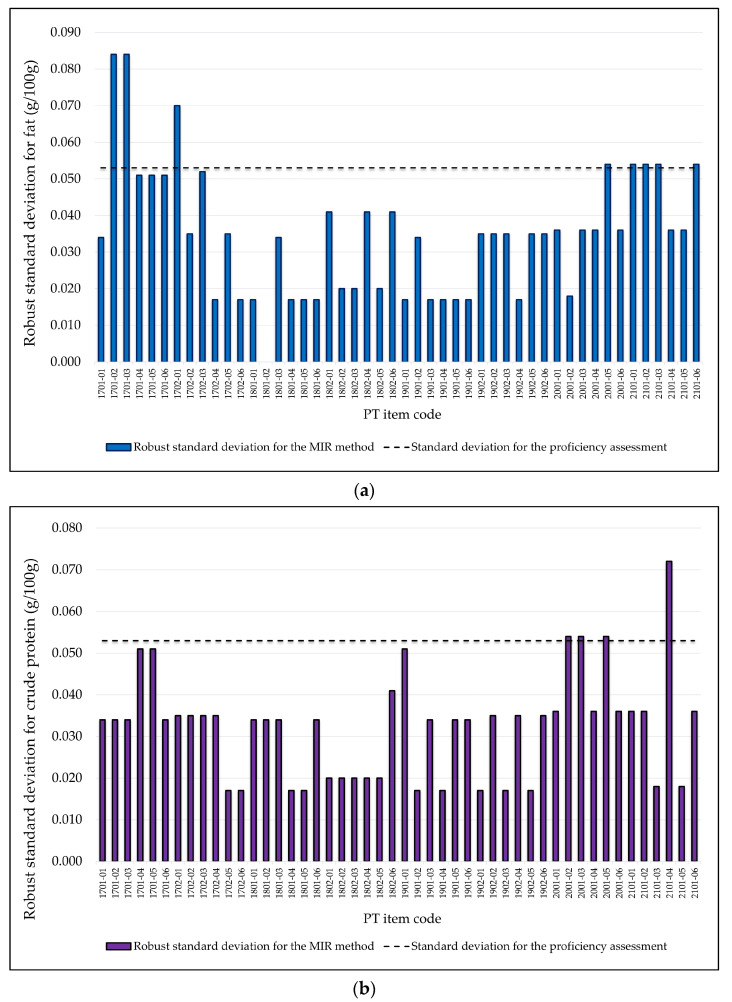
Robust standard deviation of the participants who used the MIR instrumental method in the PT rounds held between 2017 and 2021 compared to the standard deviation proposed for future PT. (**a**) Fat in raw milk; (**b**) crude protein in raw milk.

According to the criterion based on the instrumental error $e_{{\overset{¯}{x}}_{i}}$ (Equation (9)) and on these new precision and accuracy indexes [44], for a sample analyzed in duplicate through the instrumental MIR method, σ^ would be equal to 0.053 g fat or crude protein/100 g milk. In order to evaluate the applicability of this target to future PT rounds, the robust standard deviation corresponding to each PT round of the participants who used the MIR method were calculated through the robust scale estimator *Qn* [7]. The results obtained have been displayed in Figure 3. It can be observed that for fat (Figure 3a), 85.4% of the PT items showed a standard deviation equal to or lower than 0.053 g fat/100 g milk, while 10.4% of them presented standard deviation values equal to 0.054 g fat/100 g milk. On the other hand, with regard to crude protein (Figure 3b), 91.7% of the PT items obtained values equal to or lower than 0.053 g crude protein/100 g milk, while 6.3% of them showed values equal to 0.054 g crude protein/100 g milk. Updating the precision indexes for the MIR method [44] would imply a reduction of σ^ = 0.075 to σ^ = 0.053 g/100 g to be applied to future PT rounds. When this value is compared against the robust standard deviation of the participants’ results corresponding to most of the PT items, it can be seen that the whole set of laboratories has the capacity to operate at values below the new precision indexes. Nevertheless, it should be taken into account that this reduction of σ^ could imply an increase in the usage of z’-score for the MIR instrumental method (because of the lower value required to meet the uncertainty criterion), so that, based on the data in Table 3, z’-score would have to be used in 25.0% and 12.5% of the PT rounds for fat and crude protein, respectively.

#### 3.2.2. The Röse Gottlieb Method (to Determine Fat Content)

The standard ISO 1211:2010(E) [28], which has been used as the reference to establish the σ^ value, as already mentioned in Section 2.3.2, has been recently replaced by the standard ISO 23318:2022 [45], which is applicable to a range of dairy products. Nevertheless, with regard to the matrix of fluid milk, the precision indexes for the analytical methods remain to be those set out by the standard ISO 1211:2010 [28]. Consequently, no changes are proposed for future PT with regard to the σ^ value to be used for this analytical method (σ^= 0.017 g).

#### 3.2.3. The Gerber Method (to Determine Fat Content)

As already described in Section 2.3.3, the precision value set out by the standard AOAC Official Method 2000.18 [40] was considered. The precision indexes for the AOAC standard were reviewed and kept unchanged.

Even though there are other two standards that could be considered by the participating laboratories, namely ISO 19662:2018 [36] and NCh 1016/1. Of 1998 [46], in both cases, they would obtain σ^ values that are lower or just equal to those based on the AOAC Official Method 2000.18 [40]. It is therefore suggested to keep the value of σ^ at 0.050 g fat/100 g milk.

#### 3.2.4. The Kjeldahl Method (to Determine the Crude Protein Content)

As already described in Section 2.3.4, the precision value set out by the ISO standard 8968, which comprises two parts [29,30], was applied to the Kjeldahl method. No more recent amendments to the said standards have been approved; therefore, the value that had been previously established for σ^ is suggested to remain invariable (σ^= 0.015 g).

## 4. Conclusions

According to the results obtained from this research, it can be concluded that the design for the assignment of a reference value to the PT rounds that were held by the Metrology Division of the LACM^®^ between 2017 and 2021 to determine fat and crude protein in raw milk achieves a high degree of compliance with the criterion set out by the ISO 13528:2022 standard [7] for the calculation of the standard uncertainty of the assigned value. There is therefore no need to incorporate it into the evaluation of the performance of the participants who used the MIR instrumental method (no z’-score is required) and who represent 93% of the participating laboratories. We would suggest establishing, to the possible extent and according to availability, a maximum standard uncertainty value of the CRMs with regard to fat and to crude protein at 0.018 g/100 g milk and 0.021 g/100 g milk, respectively, which would maintain the uncertainty criterion of the assigned value.

Based on the results that have been obtained, it can be considered that the design for the assignment of the reference value described herein is appropriate for PT on a small number of participants, as it allows the obtainment of independently assigned values that are generally compatible with the results obtained by the participants. Furthermore, these reference values would also be traceable through CRM calibration assays by a wider set of expert laboratories.

The design could be applied to other PT as long as the analytical methods are previously verified, the uncertainty sources are identified and the required experimental design is determined, so that the standard uncertainty criterion of the assigned value set out by the ISO standard 13528:2022 [7] is met.

For future PT rounds, we would suggest taking into account any possible updates on the precision and accuracy indexes of the Mid-Infrared Spectroscopy method according to the standard ISO 8196-3 [44], so that the standard deviation for proficiency assessment can be established, while taking into account the replacement of the current value 0.075 g/100 g milk with 0.053 g/100 g milk, which could result in a larger proportion of PT items being evaluated based on their z’-scores at around 25.0% for fat and 12.5% for crude protein, according to the standard uncertainty of the assigned value that has been determined through the present research.

## Figures and Tables

**Table 1:** Experimental design for the assignment of reference values for PT on fat and crude protein content in raw milk.

Session 1			Session 2			Session 3		
PT Item	Vial	Replica	PT Item	Vial	Replica	PT Item	Vial	Replica
1	1	1	1	2	1	1	3	1
2	1	1	2	2	1	2	3	1
3	1	1	3	2	1	3	3	1
4	1	1	4	2	1	4	3	1
5	1	1	5	2	1	5	3	1
6	1	1	6	2	1	6	3	1
	CRM	1		CRM	1		CRM	1
		2			2			

**Table 2:** PT rounds for fat and crude protein in raw milk held by the PT supplier between 2017 and 2021 and identification of the CRMs used.

PT Code	Date	CRM Code	CRM Producer
EA GP LC 1701	April 2017	m-0135	muva (Kempten, Germany)
EA GP LC 1702	October 2017	m-0138	
EA GP LC 1801	May 2018	m-0140	
EA GP LC 1802	November 2018	m-0140	
EA GP LC 1901	June 2019	m-0143	
EA GP LC 1902	October 2019	m-0143	
EA GP LC 2001	October 2020	RM CP L MR M 84	DRRR (Kempten, Germany)
EA GP LC 2101	October 2021	RM CP L MR M 93	

**Table 3:** Combined standard uncertainty of the assigned value (and its components) regarding fat and crude protein in raw milk of the PT rounds held between 2017 and 2021.

PT Identification Code	Measurand	Standard Uncertainty in the CRM Certificate $u_{CRM}$ (g/100 g Milk)	Standard Uncertainty of the CRM Analysis $u_{{\bar{x}}_{CRM}}$ (g/100 g Milk)	Standard Uncertainty of the PT Item $u_{{\bar{x}}_{PTI}}$ (g/100 g Milk)	Combined Standard Uncertainty of the Assigned Value $u_{\hat{x}}$ (g/100 g Milk)
EA GP LC 1701	Fat	0.0070	0.0090	0.0102	0.0153
EA GP LC 1702		0.0050	0.0020	0.0072	0.0090
EA GP LC 1801		0.0080	0.0010	0.0083	0.0116
EA GP LC 1802		0.0080	0.0040	0.0075	0.0117
EA GP LC 1901		0.0180	0.0030	0.0112	0.0214
EA GP LC 1902		0.0180	0.0040	0.0059	0.0194
EA GP LC 2001		0.0090	0.0040	0.0036	0.0105
EA GP LC 2101		0.0110	0.0030	0.0063	0.0130
EA GP LC 1701	Crude protein	0.0050	0.0071	0.0079	0.0117
EA GP LC 1702		0.0060	0.0024	0.0029	0.0071
EA GP LC 1801		0.0130	0.0048	0.0034	0.0143
EA GP LC 1802		0.0130	0.0080	0.0031	0.0156
EA GP LC 1901		0.0110	0.0051	0.0046	0.0130
EA GP LC 1902		0.0110	0.0032	0.0024	0.0117
EA GP LC 2001		0.0210	0.0025	0.0015	0.0212
EA GP LC 2101		0.0110	0.0020	0.0026	0.0115

## Data Availability

Restrictions apply to the availability of these data. Data was obtained from the Metrology Division of the Laboratory for Measurement Quality Assurance LACM^®^ and are available on request to the corresponding author with the permission of LACM^®^. The data are not publicly available due to confidentiality arrangements with PT participants.

## Appendix A

**Table A1:** Bias of the assays for fat in raw milk on the certified reference materials (CRM) in each PT round performed between 2017 and 2021.

PT Code	$\mathrm{Average}\;{\overset{̿}{x}}_{CRM}$ (g/100 g Milk)	CRM Assigned Value $c_{CRM}$ (g/100 g Milk)	Bias $\left({\overset{̿}{x}}_{CRM}-c_{CRM}\right)$ (g/100 g Milk)
EA GP LC 1701	3.475	3.477	−0.002
EA GP LC 1702	3.524	3.525	−0.001
EA GP LC 1801	3.485	3.474	0.011
EA GP LC 1802	3.482	3.474	0.008
EA GP LC 1901	3.511	3.498	0.013
EA GP LC 1902	3.478	3.498	−0.020
EA GP LC 2001	3.590	3.594	−0.004
EA GP LC 2101	3.621	3.614	0.007

**Table A2:** Bias of the assays for crude protein in raw milk on the certified reference materials (CRM) in each PT round performed between 2017 and 2021.

PT Code	$\mathrm{Average}\;{\overset{̿}{x}}_{CRM}$ (g/100 g Milk)	CRM Assigned Value $c_{CRM}$ (g/100 g Milk)	Bias $\left({\overset{̿}{x}}_{CRM}-c_{CRM}\right)$ (g/100 g Milk)
EA GP LC 1701	3.386	3.392	−0.006
EA GP LC 1702	3.481	3.473	0.008
EA GP LC 1801	3.503	3.502	0.001
EA GP LC 1802	3.502	3.502	0.000
EA GP LC 1901	3.573	3.563	0.010
EA GP LC 1902	3.546	3.563	−0.017
EA GP LC 2001	3.452	3.431	0.021
EA GP LC 2101	3.391	3.368	0.023
